# Clarity AD: Asian regional analysis of a phase III trial of lecanemab in early Alzheimer's disease

**DOI:** 10.1016/j.tjpad.2025.100160

**Published:** 2025-04-05

**Authors:** Christopher Chen, Sadao Katayama, Jae-Hong Lee, Jun-Young Lee, Masaki Nakagawa, Kentaro Torii, Tomoo Ogawa, Amitabh Dash, Michael Irizarry, Shobha Dhadda, Michio Kanekiyo, Steve Hersch, Takeshi Iwatsubo

**Affiliations:** a Memory Aging and Cognition Center, Department of Pharmacology, Yong Loo Lin School of Medicine, National University of Singapore, Singapore; b Katayama Medical Clinic, Kurashiki, Japan; c Department of Neurology, University of Ulsan College of Medicine, Asan Medical Center, Seoul, Korea; d Department of Psychiatry, Seoul National University, Seoul, Korea; e Eisai Co., Ltd., Tokyo, Japan; f Eisai Singapore Pte Ltd., Singapore; g Eisai Inc., Nutley, NJ, USA; h Department of Neuropathology, Graduate School of Medicine, University of Tokyo, Japan; i National Center of Neurology and Psychiatry, Tokyo, Japan

**Keywords:** Alzheimer's disease, Lecanemab, disease modification

## Abstract

**Background:**

Across Asia, Alzheimer's disease prevalence is expected to rise dramatically due to, among other factors, rapidly aging populations. Alzheimer's disease pathology is triggered by the accumulation of soluble and insoluble aggregated Aβ peptides (oligomers, protofibrils, and fibrils). Lecanemab is a recently approved humanized IgG1 monoclonal antibody that preferentially targets soluble aggregated Aβ species (oligomers, protofibrils), with activity at insoluble fibrils. In the recent 18-month phase 3 Clarity AD study, lecanemab demonstrated a consistent slowing of decline in clinical (global, cognitive, functional, and quality of life) outcomes, and reduction in brain amyloid in early Alzheimer's disease. Lecanemab was well tolerated in Clarity AD, with an increase in incidence of infusion related reactions and amyloid-related imaging abnormalities (ARIA) versus placebo.

**Objectives:**

The objective of this manuscript is to present the results for the Asian region population of Clarity AD.

**Design:**

The core Clarity AD study was an 18-month, multicenter, double-blind, placebo-controlled, parallel-group study.

**Setting:**

Academic and clinical centers in Asia

**Participants:**

A total of 294 individuals with early Alzheimer's disease (i.e., mild cognitive impairment or mild Alzheimer's disease).

**Intervention:**

Eligible patients were randomized across 2 treatment groups (placebo and lecanemab 10 mg/kg biweekly) according to a fixed 1:1 schedule.

**Measurements:**

The primary efficacy endpoint in the core study was change in the Clinical Dementia Rating-Sum-of-Boxes (CDR-SB) from baseline at 18 months. Key secondary endpoints included change from baseline at 18 months in amyloid PET Centiloids (in patients participating in the amyloid PET sub-study), AD COMposite Score (ADCOMS) and AD Assessment Scale-Cognitive Subscale 14 (ADAS-Cog14). Safety was monitored throughout the study in a blinded manner by the sponsor and in an unblinded manner by an independent data safety monitoring committee.

**Results:**

Of the total of 1795 subjects randomized in Clarity AD, 294 subjects were in the Asian region (Japan:152; Korea:129; Singapore:13). The efficacy of lecanemab was consistent with the overall population. For the primary endpoint, there was a slowing of decline with lecanemab in the CDR-SB at 18 months compared to placebo in the Asian region (adjusted mean difference: -0.349; 95 % confidence intervals: -0.773, 0.076; 24 % slowing of decline). Results for the secondary efficacy endpoints also favored lecanemab versus placebo in Asians. Lecanemab was well tolerated in Asian subjects, with a safety profile in Asian subjects similar to the overall Clarity AD population. The most common adverse events of special interest were ARIA-H (lecanemab:14.4 %; placebo:16.2 %), ARIA-E (lecanemab:6.2 %; placebo:1.4 %), and infusion-related reactions (lecanemab:12.3 %; placebo:1.4 %). Incidence of adverse events leading to study drug dose interruption or withdrawal, infusion-related reactions, ARIA-E and ARIA-H was lower for the lecanemab treated group in the Asian region relative to the overall Clarity AD population. Results from quality of life and biomarker assessments in the Asia region were also generally similar to the overall Clarity AD population.

**Conclusion:**

In the Clarity AD Asian region cohort, the overall efficacy, biomarker changes and safety profile of lecanemab were consistent with the overall population, with a favorable risk-benefit profile and manageable risks. ARIA events and infusion-related reactions occurred less commonly with lecanemab in the Asian region subgroup than the overall population.

## 1 Introduction

As the global population is living longer, Alzheimer's disease (AD) is posing an increasingly significant health care issue [[1], [2], [3], [4]]. Despite efforts toward reducing the number of AD cases through educational, policy, and various public health and preventive medicine interventions, the prevalence of AD continues to grow in Asia, due to a large and ageing population. Dementia care is a significant economic burden in Asia [5]. Costs are estimated at US$185 billion, with 70 % of this amount occurring in advanced economies, which have only 18 % of the prevalence [5,6].

In the past, therapeutic agents for Alzheimer's disease dementia transiently improve symptoms but do not alter the underlying disease course [[7], [8], [9]]. Growing evidence suggests that amyloid removal slows progression of disease [10,11]. Recently, phase 3 studies have demonstrated that anti-amyloid disease modifying therapies can improve the lives of those with early AD and slow progression of the disease [[12], [13], [14], [15], [16], [17]].

Lecanemab is an anti-amyloid monoclonal antibody that binds with the highest affinity to soluble Aβ protofibrils, which are more toxic than monomers or insoluble fibrils/plaque [11,[18], [19], [20], [21], [22], [23], [24], [25], [26]]. A phase 2 study conducted in 856 participants with early Alzheimer's disease demonstrated dose and time dependent clearance of amyloid and reduction in clinical decline in 18-month clinical outcomes for lecanemab versus placebo. Lecanemab 10-mg/kg IV biweekly was identified as the optimal dosage as it was generally well-tolerated, with 9.9 % incidence (<3 % symptomatic) of amyloid-related imaging abnormalities-edema (ARIA-E; 12). In the subsequent phase 3 Clarity AD study, lecanemab demonstrated a consistent slowing of decline in clinical (global, cognitive, functional, and quality of life) outcomes, and reduction in brain amyloid in early AD [13].

Here, we present data from the Asian regional cohort of Clarity AD. This subgroup analysis includes 294 subjects enrolled in Japan, South Korea and Singapore.

## 2 Methods

### 2.1 Trial design and oversight

The overall design of Clarity AD has been previously published (13; ClinicalTrials.gov identifier: Clarity AD NCT03887455). Briefly, Clarity AD was an 18-month global, multicenter, double-blind, placebo-controlled, parallel-group study in individuals with early Alzheimer's disease. Eligible participants were randomized to placebo or lecanemab 10 mg/kg IV biweekly according to a fixed 1:1 schedule. The randomization was stratified according to clinical subgroup (mild cognitive impairment due to Alzheimer's disease or mild Alzheimer's disease dementia); presence or absence of concomitant approved Alzheimer's disease symptomatic medication at baseline (e.g., acetylcholinesterase inhibitors, memantine, or both); ApoE4 status (ie, carriers or non-carriers); and geographical region (ie, North America, Europe, or Asia). Participants had serial blood draws for plasma biomarkers during the study and could participate in three optional substudies that evaluated longitudinal changes in brain amyloid burden as measured by amyloid PET, brain tau pathology as measured by tau PET, and CSF biomarkers of Alzheimer's disease pathology. This analysis focuses on the subgroup of participants that were enrolled from Asia.

The study was conducted in accordance with International Conference on Harmonisation guidelines and ethical principles of the Declaration of Helsinki. All participants provided written informed consent, and the study was approved by the institutional review board or independent ethics committee at each center. The safety data was monitored by an independent Data Safety Monitoring Board (DSMB) consisting of experts in Alzheimer's disease and statistics.

### 2.2 Eligibility criteria

Clarity AD included participants aged 50 to 90 years, with either mild cognitive impairment due to Alzheimer's disease or mild Alzheimer's disease dementia based on National Institute of Aging–Alzheimer's Association (NIA-AA) criteria [[27], [28]]. Amyloid pathology was determined by positron emission tomography (PET) or cerebrospinal fluid (CSF) measurement of t-tau/ Aβ(1–42). All participants were required to have objective impairment in episodic memory with *a* ≥ 1 standard deviation below the age-adjusted mean in the Wechsler Memory Scale IV-Logical Memory (subscale) II.

### 2.3 Endpoints

Change from baseline to 18 months in the Clinical Dementia Rating-Sum-of-Boxes (CDR-SB; [29]) was the primary efficacy endpoint. Key secondary endpoints included change from baseline at 18 months in amyloid PET using Centiloids (with either florbetaben, florbetapir, or flutemetamol tracers), Alzheimer's disease Assessment Scale-Cognitive Subscale 14 (ADAS-Cog14; [30]), Alzheimer's disease COMposite Score (ADCOMS; [31]), and Alzheimer's Disease Cooperative Study-Activities of Daily Living Scale for Mild Cognitive Impairment (ADCS-MCI-ADL; [32]). Biomarker assessments included plasma biomarkers (Aβ 42/40 ratio, p-tau181, glial fibrillary acidic protein [GFAP], and neurofilament light chain [NfL]). Health-related quality of life (HRQoL) scales utilized included the European Quality of Life–5 Dimensions (EQ-5D-5L), Quality of Life in AD (QOL-AD) and the Zarit Burden Interview (ZBI) [[33], [34], [35], [36]].

### 2.4 Statistical analysis

The statistical analysis for the overall Clarity AD study was previously published [13]. For the Asian regional cohort, all results are summarized descriptively, as the analyses for subgroups are not powered to evaluate statistical differences. Efficacy analyses were performed in the modified intention-to-treat population defined as the group of randomized participants who received at least one dose of study drug, and who had a baseline assessment and at least one post-dose primary efficacy measurement. Safety evaluations conducted in all enrolled subgroup participants included monitoring of adverse events, vital signs, physical examinations, clinical laboratory parameters, and 12-lead electrocardiograms. ARIA occurrence was monitored throughout the study by central reading of magnetic resonance imaging performed for safety monitoring. In addition, the PET substudy population was the group of participants who received at least one dose of study drug and who had participated in the PET substudy with a baseline and at least one postdose scan.

The primary analysis was based on a modified intention-to-treat principle without imputation of missing values. The primary analysis of the change from baseline at 18 months in CDR-SB was performed to compare lecanemab versus placebo using a mixed model for repeated measures. Subgroup analyses based on randomization stratification were conducted and those conducted for the Asian region are presented.

## 3 Results

### 3.1 Participants

Baseline characteristics were generally similar across treatment groups (Table 1). Demographic and disease-related baseline characteristics in the Asia region population were generally similar to those of the overall population, except for the lower mean body weight and slightly higher rates of MCI due to AD and global CDR=0.5 in the Asian regional population. Overall, 294 subjects from Japan, South Korea and Singapore were enrolled in Clarity AD: 152 from Japan (placebo:64, lecanemab:88), 129 from South Korea (placebo:77, lecanemab: 52), and 13 from Singapore (placebo:7, lecanemab:6) (Table 1).

**Table 1:** Baseline Characteristics.

Mean (SD), if not specified	Overall		Asia Region	
	Placebo (*N* = 897)	Lecanemab (*N* = 898)	Placebo (*N* = 148)	Lecanemab (*N* = 146)
**Age, years**	71.1 (7.8)	71.4 (7.9)	69.8 (7.6)	70.3 (8.1)
**Female, n ( %)**	476 (53.1)	462 (51.4)	86 (58.1)	85 (58.2)
**Country**				
**Japan**	64 (7.1)	88 (9.8)	64 (43.2)	88 (60.3)
**South Korea**	77 (8.6)	52 (5.8)	77 (52.0)	52 (35.6)
**Singapore**	7 (0.8)	6 (0.7)	7 (4.7)	6 (4.1)
**Weight, kg**	70.8 (15.0)	71.6 (15.5)	58.0 (10.2)	56.6 (10.3)
**Years since diagnosis**	1.3 (1.5)	1.4 (1.5)	1.4 (1.3)	1.5 (1.5)
**Years since symptom onset**	4.2 (2.5)	4.1 (2.4)	3.8 (2.2)	3.7 (2.3)
**MCI due to AD, n ( %)**	555 (61.9)	552 (61.5)	104 (70.3)	102 (69.9)
**ApoE4 Status, n ( %)**				
**Noncarrier**	286 (31.9)	278 (31.0)	42 (28.4)	40 (27.4)
**Carrier**	611 (68.1)	620 (69.0)	106 (71.6)	106 (72.6)
***Heterozygous***	478 (53.3)	479 (53.3)	76 (51.4)	81 (55.5)
***Homozygous***	133 (14.8)	141 (15.7)	30 (20.3)	25 (17.1)
**Global CDR Score = 0.5**	706 (80.7)	694 (80.8)	136 (91.9)	142 (96.6)
**Global CDR Score = 1.0**	169 (19.3)	165 (19.2)	12 (8.1)	5 (3.4)
**CDR-SB, mean (SD)**	3.22 (1.343)	3.17 (1.340)	2.81 (1.185)	2.76 (0.999)
**PET Centiloids, mean (SD)**	75.03 (41.82)	77.92 (44.84)	76.42 (37.28)	82.21 (33.56)
**ADAS-Cog14, mean (SD)**	24.37 (7.561)	24.45 (7.082)	26.79 (6.809)	25.87 (5.467)
**ADCOMS, mean (SD)**	0.400 (0.15)	0.398 (0.15)	0.370 (0.13)	0.376 (0.12)
**ADCS-MCI-ADL**	40.9 (6.89)	41.2 (6.61)	40.3 (6.97)	39.9 (5.86)
**MMSE, mean (SD)**	25.6 (2.23)	25.5 (2.19)	25.4 (2.08)	25.2 (2.12)

LEC-10BW, Lecanemab 10 mg/kg biweekly; PBO, Placebo; AChEI, Acetylcholinesterase inhibitor; ApoE4, Apolipoprotein E4; CDR, Clinical Dementia Rating Scale; MMSE, Mini-Mental State Exam; PET, Positron emission tomography; SD, standard deviation.

### 3.2 Efficacy

**Fig. 1:**
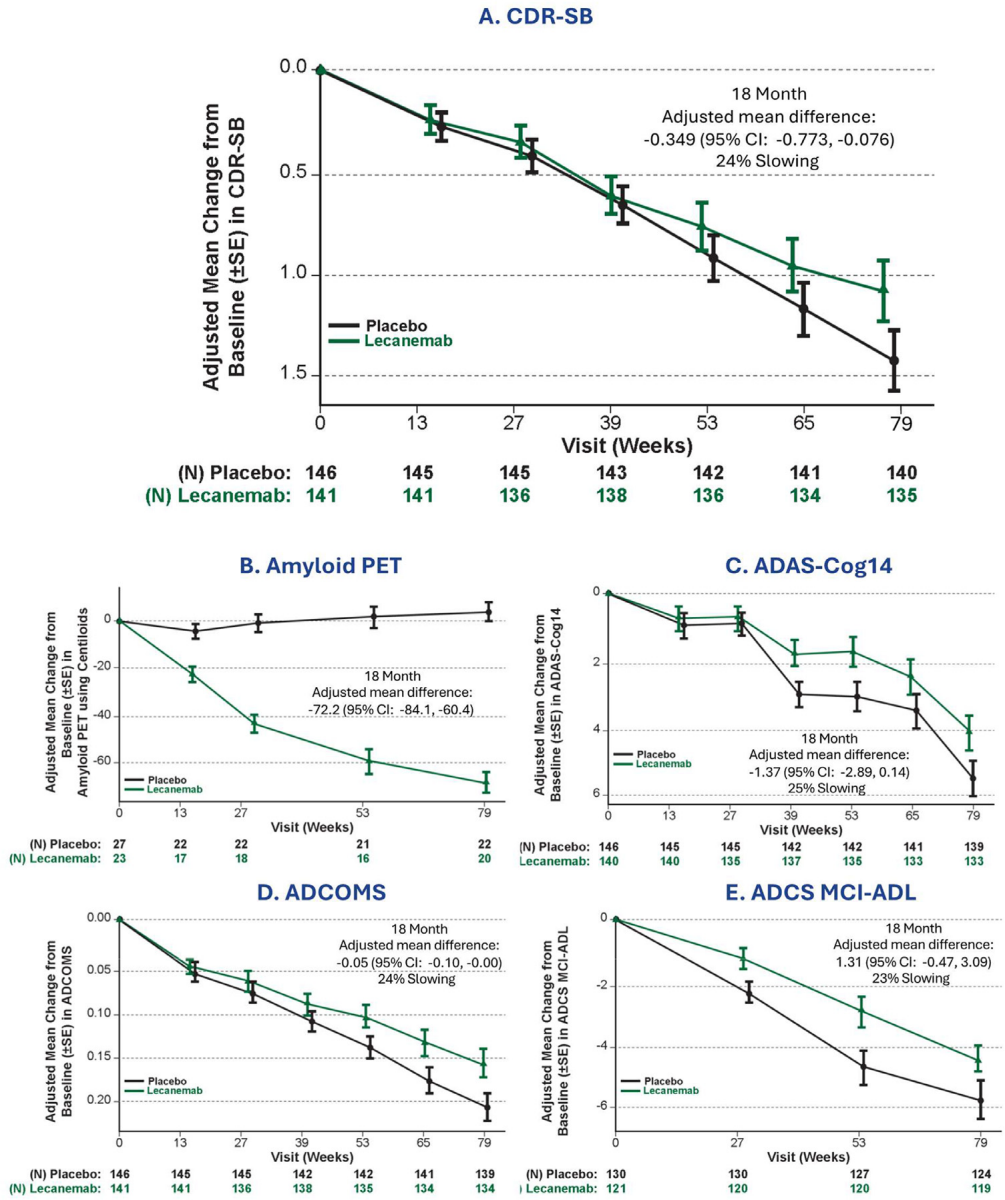
Efficacy Assessment Results for A. CDR-SB (Primary Endpoint), B. Amyloid PET, C. ADAS-Cog14, D. ADCOMS, and E. ADCS MCI-ADL.

Results for the primary endpoint analysis (CDR-SB) are shown in Fig. 1. There was a 24 % slowing of decline with lecanemab in CDR-SB at 18 months compared to placebo in the Asian regional population, consistent with the overall population. At 18 months, the adjusted mean difference was −0.349 (95 % CI: −0.773, −0.076) for the CDR-SB assessment. Secondary endpoint results were generally consistent with the CDR-SB (Fig. 1). There was a slowing of decline with lecanemab in all key secondary endpoints at 18 months compared to placebo in the Asian regional population, consistent with the overall population. At 18 months, adjusted mean differences at 18 months were −72.2 (95 % CI: −84.1, −60.4) for Amyloid PET. Adjusted mean change from baseline at 18 months showed 25 % less decline for ADAS-Cog14 (difference: −1.37(95 % CI: −2.89, 0.14)) and 24 % for ADCOMS (difference: −0.05 (95 % CI: −0.10, −0.00)), and 23 % for ADCS MCI-ADL (difference: 1.31 (95 % CI: −0.47, 3.09)).

CDR-SB, Clinical Dementia Rating Scale sum of boxes. SE, Standard error. CI, Confidence interval. PET, Positron emission tomography. ADAS-Cog14, 14-item Alzheimer's Disease Assessment Scale–cognitive subscale; ADCOMS, Alzheimer's Disease Composite Score. ADCS-ADL-MCI, Alzheimer's Disease Cooperative Study-Activities of Daily Living Scale for Mild Cognitive Impairment.

**Fig. 2:**
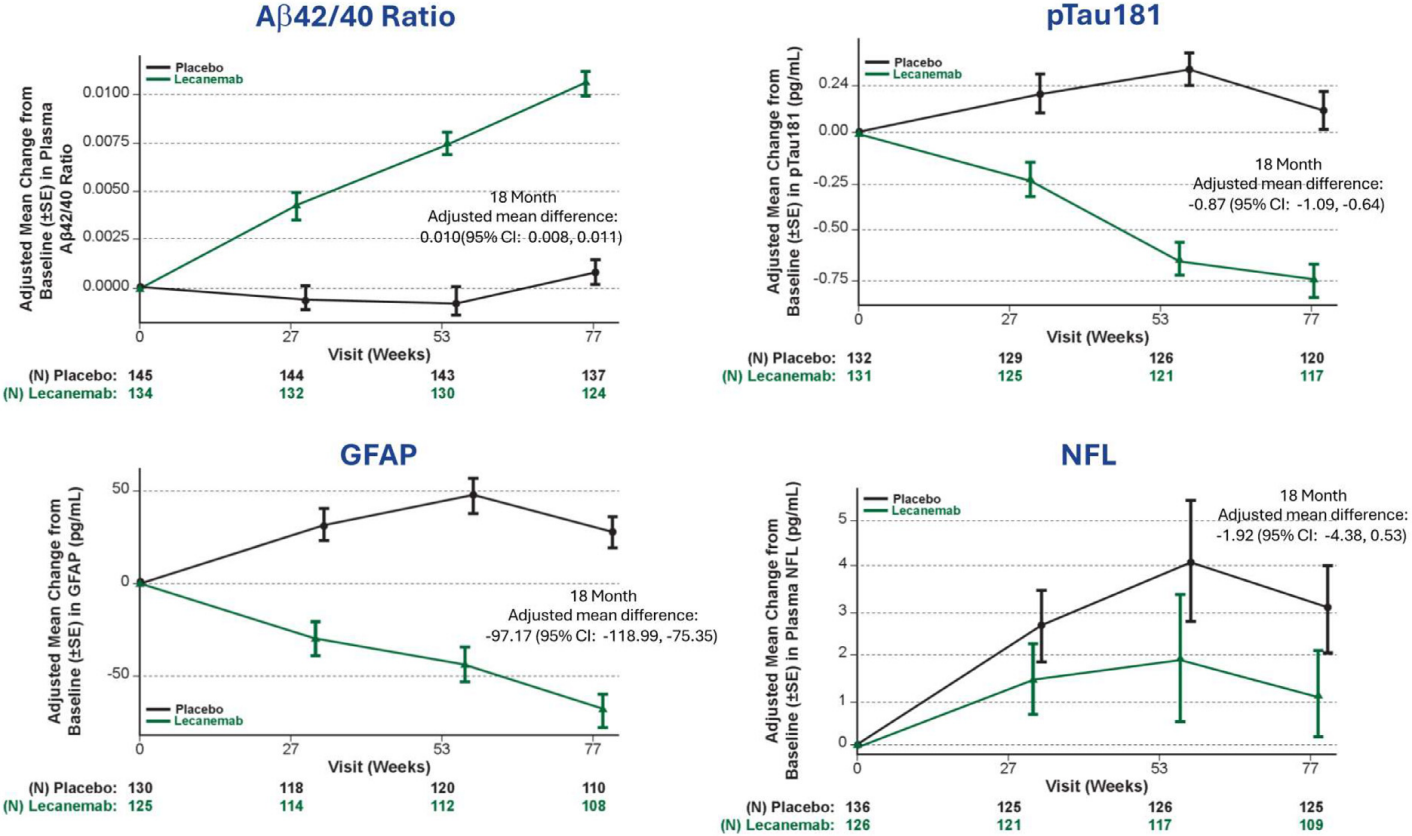
Plasma Biomarker Results.

Plasma biomarker results are highlighted in Fig. 2. There was a positive effect on plasma biomarkers of amyloid, tau and neuroinflammation at 18 months compared to placebo in the Asian population consistent with the overall population. Adjusted mean differences at 18 months were 0.010 (95 % CI:0.008, 0.011) for Aβ42/40 ratio, −0.87 (95 % CI: −1.09, −0.64) for pTau181, −97.17 (95 % CI: −118.99, −75.35) for GFAP, and −1.92 (95 % CI: −4.38, 0.53) for NfL.

Aβ, Amyloid β. p-tau, phosphorylated tau. GFAP, glial fibrillary acidic protein. NFL, Neurofilament light chain. SE, Standard error. CI, Confidence interval.

### 3.3 Safety

The overall incidence of adverse events (AEs) was similar among treatment groups (Table 2). The incidence of AEs and serious AEs were similar to those in the overall population. Most of AEs were mild to moderate. In lecanemab, the incidence of severe AEs, treatment-related AEs, AEs leading to dose interruption or withdrawal were lower than that in the overall population. The incidence of AEs leading to infusion interruption was higher for lecanemab (3.4 %) than that in the overall population (2.4 %), however, the incidence was similar between placebo and lecanemab (2.7 % vs 3.4 %).

**Table 2:** Adverse Events.

	Overall		Asia Region	
	Placebo (*N* = 897)	Lecanemab (*N* = 898)	Placebo (*N* = 148)	Lecanemab (*N* = 146)
**Adverse event (AE)**	735 (81.9)	798 (88.9)	118 (79.7)	122 (83.6)
*severe*	*61 (6.8)*	*67 (7.5)*	5 (3.4)	4 (2.7)
**Treatment-related AE**	197 (22.0)	401 (44.7)	33 (22.3)	43 (29.5)
**Serious AE**	101 (11.3)	126 (14.0)	19 (12.8)	19 (13.0)
*Death*	*7 (0.8)*	*6 (0.7)*	*0*	*0*
**AE leading to study drug dose adjustment**	95 (10.6)	219 (24.4)	9 (6.1)	16 (11.0)
*leading to study drug withdrawal*	*28 (3.1)*	*64 (7.1)*	2 (1.4)	1 (0.7)
*leading to study drug dose interruption*	*71 (7.9)*	*175 (19.5)*	7 (4.7)	15 (10.3)
*leading to infusion interruption*	*11 (1.2)*	*22 (2.4)*	4 (2.7)	5 (3.4)
**AEs of special interest**				
ARIA-E	15 (1.7)	113 (12.6)	2 (1.4)	9 (6.2)
ARIA-H	81 (9.0)	155 (17.3)	24 (16.2)	21 (14.4)
*Infusion-related reactions*	*66 (7.4)*	*237 (26.4)*	2 (1.4)	18 (12.3)

ARIA, amyloid-related imaging abnormalities. ARIA-E, ARIA with edema. ARIA-H, ARIA with hemosiderin deposits, cerebral microhemorrhage, superficial siderosis, and intracerebral hemorrhage/macrohemorrhage.

The most common adverse effects of special interest were ARIA-H (lecanemab:14.4 %; placebo:16.2 %), ARIA-E (lecanemab:6.2 %; placebo:1.4 %), and infusion-related reactions (lecanemab:12.3 %; placebo:1.4 %). The incidence of ARIA-E in lecanemab was higher than in placebo (6.2 % vs 1.4 %), but lower in the Asian regional population than that in the overall population for lecanemab (12.6 %). All the ARIA-E were asymptomatic, mild to moderate in radiographic severity, and resolved over time. Most of ARIA-E occurred within 6 months of treatment, which was consistent with the overall population. The incidence of ARIA-H was slightly lower for lecanemab than placebo in Asia-region population (14.4 % vs 16.2 %). The incidence of ARIA-H for lecanemab in the Asian region was lower compared to the overall population (14.4 % vs 17.3 %). The incidence of isolated ARIA-H was similar between lecanemab and placebo. Almost all ARIA-H in lecanemab were asymptomatic and radiographically mild to moderate. The incidence of infusion-related reactions (12.3 %) was lower than that in the overall population for lecanemab (26.4 %;placebo: 7.4 %). All the infusion-related reactions in lecanemab were mild or moderate and grade 1 or 2.

### 3.4 Health-related quality of life

**Fig. 3:**
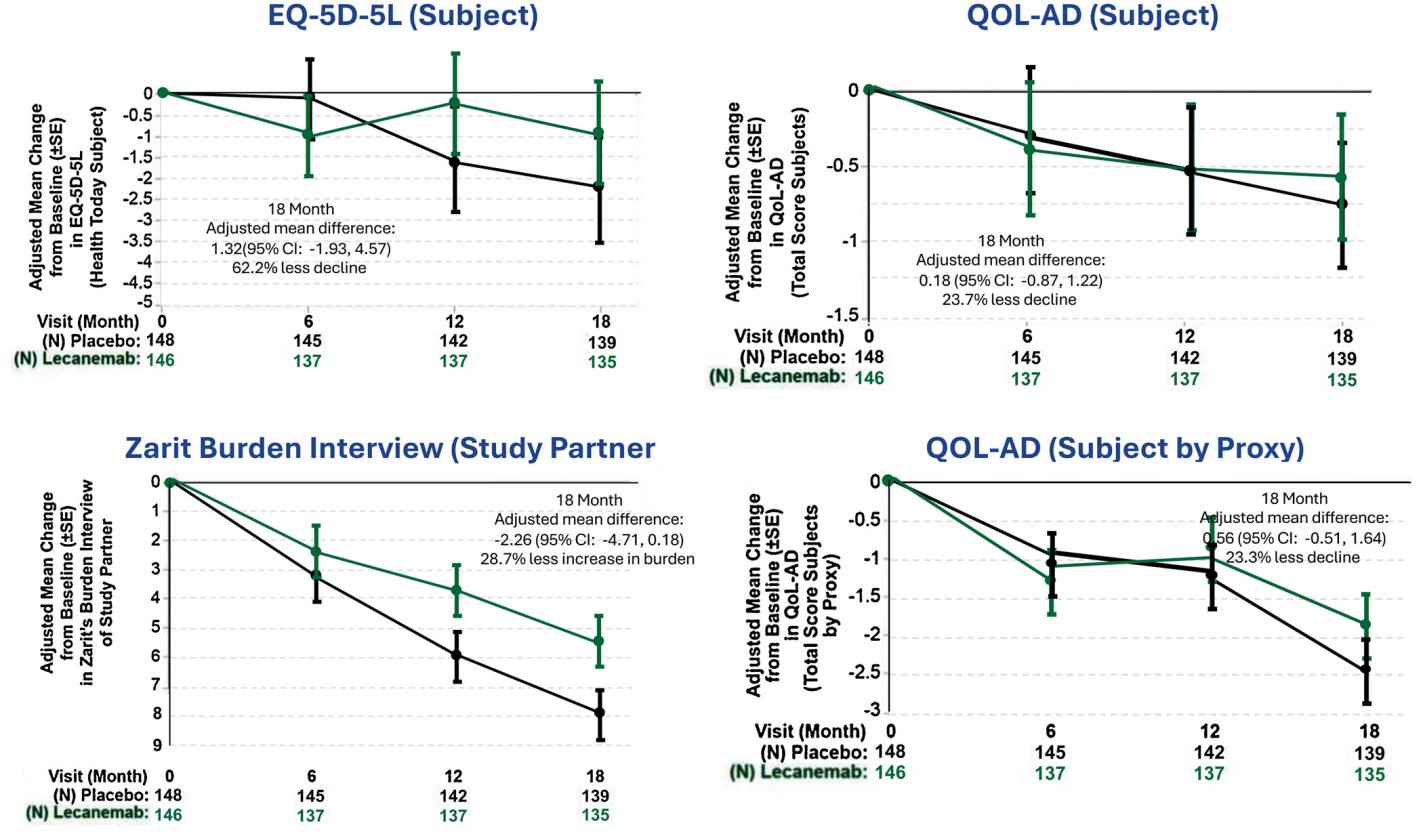
Health-Related Quality of Life Results.

At month 18, adjusted mean change from baseline in EQ-5D-5L (health today) and QOL-AD (total score) by subject showed 62.2 % and 23.7 % less decline for lecanemab vs placebo, respectively (Fig. 3**)**. Adjusted mean change from baseline at 18 months was 1.32 (95 % CI: −1.93, 4.57) for EQ-5D-5L Health Today (subject) and 0.18 (95 % CI: −0.87, 1.22) for QOL-AD Total Score (subject). QOL-AD (total score) rated by study partner as proxy resulted in 23.3 % less decline for lecanemab vs placebo, with an adjusted mean difference at 18 months from baseline of 0.56 (95 % CI: −0.51, 1.64). ZBI adjusted mean change from baseline at 18 months (−2.26 [95 % CI: −4.71, 0.18]) resulted in 28.7 % less increase of care partner burden for lecanemab vs placebo. Individual HRQoL test items and dimensions also showed lecanemab benefit.

EQ-5D-5L, European Quality of Life–5 Dimensions 5 Level version. QOL-AD, Quality of Life in Alzheimer's Disease. SE, Standard error. CI, Confidence interval.

## 4 Discussion

In this report, we present the data of the results for the Asian regional cohort from Clarity AD, which included participants from Japan, Korea, and Singapore. Overall, lecanemab was as efficacious and safe with manageable risks as in the overall population [13], with a favorable risk-benefit profile in the Asian population. Specifically, there was a slowing decline with lecanemab in CDR-SB and all key secondary endpoints at 18 months compared to placebo in the Asian population, which was consistent with the overall population (**Table S1**). In addition, there was a brain amyloid reduction in amyloid PET and a positive effect on plasma biomarkers of AT(N) with lecanemab at 18 months compared to PBO in all the Asia region population.

Lecanemab was generally well tolerated in Asian subjects, with a safety profile in Asian subjects consistent to the global Clarity AD trial. The incidence of adverse events leading to study drug dose interruption or withdrawal, and AEs of special interest such as infusion-related reactions, ARIA-E and ARIA-H tended to be lower in the Asia region population than those of the overall population [13]. The mechanism for the lower frequency of adverse events in the Asia region is not clear, especially given the ApoE4 carrier frequency was similar in the Asia region (72.6 %) to that in the overall population (69.0 %). Body weight was lower in the Asia region relative to the overall population; a previous analysis showed that although body weight had an impact on lecanemab exposure, the differences in exposure were not clinically meaningful (data on file, Eisai Inc.). A lower prevalence of cerebral amyloid angiopathy (CAA) in the Asia region may be a possible explanation, but available research is limited and published data have mixed results [[37], [38], [39], [40]]. Most of the ARIA-H observed in this population was isolated ARIA-H or microhemorrhage, which was different than the overall population where most ARIA-H was concurrent with ARIA-E. This observation may reflect a greater relative presence of vascular disease, particularly small vessel disease in the Asian population besides CAA. Cerebral small vessel disease is associated with isolated microhemorrhage. Another possible mechanism for lower ARIA rates may be differences in amyloid burden relative to the overall population, although that association has not been observed with lecanemab [41]. However, amyloid was similar at baseline (Asia region: 82.2 and overall:77.9) and 18 months (Asia region:13.3 and overall:23.0) for the Asia region and overall populations. In the Asian population, lecanemab was associated with a relative preservation of HRQoL and less increase in caregiver burden, with consistent benefits seen across different quality of life scales.

Although similar, the frequency of ApoE4 carriers in the Asian region was slightly higher relative to the overall Clarity AD population. This may be consistent with prior findings that APOE4 alleles are less common in Japanese populations but have been associated with increased risk of developing AD relative to other ethnic subgroups [42].

Published data of anti-amyloid antibodies for the treatment of AD in Asia region populations are limited in the literature. There are two published reports on Japanese subgroup analyses for aducanumab and donanemab [[43], [44]]. For aducanumab, the efficacy, safety, biomarker, and PK profiles were reported as consistent between the Japanese subgroup and the overall population from the EMERGE and ENGAGE clinical trials [43]. The efficacy endpoint results were mixed, with a positive treatment effect of aducanumab observed in the EMERGE trial, but not in ENGAGE [41]. For donanemab, overall efficacy and safety of donanemab in Japanese participants were as similar to the global TRAILBLAZER-ALZ 2 study population [44]. Of note, the results for ARIA-E and ARIA-H vary among the available anti-amyloid antibody Asian region subgroup data. For example, the Japanese subpopulation analysis from the TRAILBLAZER-2 study reported a higher incidence of any ARIA with donanemab compared with placebo (40.0 % vs. 14.0 % in the Japanese subpopulation), similar to results from the overall population (36.8 % vs. 14.9 %; 43). This is in contrast to data available for aducanumab in the Japanese subpopulation [42] and for lecanemab in the Asian subpopulation, where the frequency of ARIA events was lower in the Asian subpopulations relative to the overall populations [13], [14], [15], [45]. However, due to the lack of prospective, comparative data among the anti-amyloid antibodies, these cross-study comparisons should be interpreted with appropriate caution.

There were some limitations in this study. This study included only 18 months of treatment and efficacy/safety beyond that timeframe is unknown. However, there is an open-label extension ongoing for overall Clarity AD and phase 2 open-label extension data are available/supportive [12]. In addition, this a subgroup analysis of 294 patients. Subgroup analyses can result in multiplicity concerns. Formal statistical analysis was not possible, however, data is generally consistent with results observed with larger overall Clarity AD population [23]. Heterogeneity across East Asian populations may also limit generalization to the rest of Asia and Asian populations elsewhere.

In summary, in this Asia region cohort, the overall efficacy, biomarker changes & safety profile of lecanemab were consistent with the overall population, with a favorable risk-benefit profile including with manageable risks. ARIA events and infusion-related reactions occurred less commonly with lecanemab in the Asia region subgroup than the overall population.

## Funding

This study was funded by Eisai Inc. and Biogen.

## CRediT authorship contribution statement

**Christopher Chen:** Writing – review & editing, Writing – original draft, Investigation, Formal analysis, Conceptualization. **Sadao Katayama:** Writing – review & editing, Writing – original draft, Methodology, Investigation, Formal analysis, Conceptualization. **Jae-Hong Lee:** Writing – review & editing, Writing – original draft, Methodology, Investigation, Conceptualization. **Jun-Young Lee:** Writing – review & editing, Writing – original draft, Methodology, Investigation, Formal analysis, Conceptualization. **Masaki Nakagawa:** Writing – review & editing, Writing – original draft, Formal analysis. **Kentaro Torii:** Writing – review & editing, Writing – original draft, Formal analysis. **Tomoo Ogawa:** Writing – review & editing, Writing – original draft, Formal analysis. **Amitabh Dash:** Writing – review & editing, Writing – original draft, Formal analysis, Conceptualization. **Michael Irizarry:** Writing – review & editing, Writing – original draft, Methodology, Formal analysis, Conceptualization. **Shobha Dhadda:** Writing – review & editing, Writing – original draft, Methodology, Investigation, Formal analysis, Data curation, Conceptualization. **Michio Kanekiyo:** Writing – review & editing, Writing – original draft, Methodology, Investigation, Formal analysis. **Steve Hersch:** Writing – review & editing, Writing – original draft, Conceptualization. **Takeshi Iwatsubo:** Writing – review & editing, Writing – original draft, Methodology, Investigation, Formal analysis, Conceptualization.

## Declaration of competing interest

The authors declare the following financial interests/personal relationships which may be considered as potential competing interests: Christopher Chen reports a relationship with Eisai Inc and Cerecin that includes: personal consulting fees.

Sadao Katayama has no known competing financial interests or personal relationships that could have appeared to influence the work reported in this paper.

JH Lee reports has no known competing financial interests or personal relationships that could have appeared to influence the work reported in this paper.

Jun-Young Lee has no known competing financial interests or personal relationships that could have appeared to influence the work reported in this paper.

Masaki Nakagawa reports a relationship with Eisai that includes: employment.

Kentaro Torii reports a relationship with Eisai that includes: employment.

Tomoo Ogawa reports a relationship with Eisai that includes: employment.

Amitabh Dash reports a relationship with Eisai that includes: employment.

Michael Irizarry reports a relationship with Eisai that includes: employment.

Shobha Dhadda reports a relationship with Eisai that includes: employment.

Michio Kanekiyo reports a relationship with Eisai that includes: employment.

Steve Hersch reports a relationship with Eisai that includes: employment.

Takeshi Iwatsubo reports a relationship with Eisai Inc that includes: personal consulting fees.

## Appendix Supplementary materials

Image, application 1
