# Supplementary material for: Clarity AD: Asian regional analysis of a phase III trial of lecanemab in early Alzheimer's disease
Source: J Prev Alzheimers Dis. 2025 Apr 5;12(5):100160. doi: 10.1016/j.tjpad.2025.100160 (PMC12184022; doi:10.1016/j.tjpad.2025.100160)
Supplement: Supplementary file 1 [file mmc1.docx]

**Table S1.** Primary and Secondary Endpoints: Results for Overall Population and Asian Region Subgroup in Clarity AD

|  | **Overall Population**  **(N=1734)** | **Asian Region Subgroup**  **(N=287)** |
| --- | --- | --- |
| **CDR-SB**  Adjusted mean difference vs placebo (percent difference)  95% confidence interval | −0.45 (27%)  −0.67, −0.23 | -0.349 (24%) -0.773, -0.076 |
| **ADAS-Cog14**  Adjusted mean difference vs placebo (percent difference)  95% confidence interval | −1.44 (26%)  −2.27 to −0.61 | -1.37 (25%) -2.89, 0.14 |
| **ADCOMS**  Adjusted mean difference vs placebo (percent difference)  95% confidence interval | −0.050 (24%)  −0.074 to −0.027 | -0.05 (24%)  -0.10, -0.00 |
| **ADCS MCI-ADL**  Adjusted mean difference vs placebo (percent difference)  95% confidence interval | 2.0 (37%)  1.2 to 2.8 | 1.31 (23%)  -0.47, 3.09 |
| **Amyloid PET***  Adjusted mean difference vs placebo, centiloids  95% confidence interval | −59.12  −62.64 to −55.60 | -72.2  -84.1, -60.4 |

*For amyloid PET analysis, the Ns for overall population and Asian region subgroup were 277 and 50, respectively.
